# Moderating Effect of Self-Esteem on the Relationship between Depression and Family Conflict Coping Strategies in the Elderly with Chronic Diseases in Korea

**DOI:** 10.3390/healthcare11182569

**Published:** 2023-09-18

**Authors:** Jae Hee Kim, Hwa-Mi Yang

**Affiliations:** Department of Nursing, Daejin University, Pocheon-si 11159, Republic of Korea; hjw9266@daejin.ac.kr

**Keywords:** self-esteem, depression, family conflict, coping strategies, elderly

## Abstract

(1) Background: The elderly with chronic diseases often experience high levels of depression, which can negatively affect their family conflict coping strategies. Additionally, as the level of depression increases, self-esteem tends to decrease. This study aims to investigate whether self-esteem plays a moderating role in the relationship between depression and family conflict coping strategies among the elderly with chronic diseases. (2) Method: The subjects were 2501 older adults with chronic diseases included in the 16th Korean Welfare Panel Study. The CES-D scale, Rosenberg Self-Esteem Scale, and the tool of Family Conflict Coping Strategies were used. (3) Results: Depression was negatively correlated with self-esteem and coping strategies for family conflict, while self-esteem was positively correlated with coping strategies for family conflict. In the multiple regression model, self-esteem had a moderating effect (F = 43.20, *p* < 0.001). This result indicated that as self-esteem increases, the negative influence of depression on family conflict coping strategies becomes weaker (β = −0.08, t = −3.04, *p* = 0.002). (4) Conclusions: When addressing family conflict coping strategies in the elderly with chronic diseases, it is crucial to focus on enhancing self-esteem. Additionally, it may be beneficial to classify the elderly into groups based on their level of self-esteem.

## 1. Introduction

The global elderly population is expected to increase, reaching 1.4 billion by 2030 and 2.1 billion by 2050 [1]. South Korea is known as one of the OECD countries with the fastest aging population. As of 2022, the proportion of the elderly population has reached 17.5% [2]. Despite the advances in medicine and science contributing to an increased lifespan, the prevalence of chronic diseases among the elderly population, including dementia, cerebrovascular diseases, and hypertension, remains as a serious social issue [3]. Particularly, elderly individuals with chronic diseases are more likely to experience negative psychological states such as depression and family conflicts [4,5].

Depression in the elderly has emerged as a crucial public health concern [6]. Depression is a prevalent mental disorder among the elderly, and the global prevalence of depression among the elderly was 28.4% [7]. Depression in the elderly is associated not only with 48–51% higher total healthcare costs compared to non-depressed elderly individuals, but also with a decline in their quality of life, increased suicide rates, and mortality due to diseases [8]. In cases where chronic diseases accompany elderly depression, this has been reported to have negative effects on treatment and prognosis [6]. Poor overall health due to chronic illnesses and emotional burden from accumulated negative experiences can lead to depression [5,7]. In older adults with a chronic illness, depression is associated with decreased self-care confidence [9]. Thus, older adults with chronic conditions need more help or support from their families and may become more dependent on them. This dependent situation can reduce the ability to cope with family conflicts because they cannot control their condition. Family plays a crucial role in supporting elderly individuals with chronic diseases [10,11]. However, elderly individuals with chronic diseases often feel burdened in sharing information about their conditions with their families and experience deteriorating family relationships [4]. Depressed people often tend to fall into negative thought patterns, which lead to self-criticism and decreased self-confidence [9], impairing their ability to cope with family conflicts. Moreover, this negative mindset may lead to the deterioration of relationships with family members.

The way conflicts within families are resolved is more critical than the conflicts themselves. Depending on the coping methods used to address conflicts, the perceived quality and satisfaction of family relationships among family members can vary [12,13]. Asian countries have a culture of collectivism, whereas Western countries such as Australia, New Zealand, the United Kingdom, and the United States tend to be more individualistic [14]. Therefore, collectivism in Asian cultures often emphasizes family-oriented support systems. In Korea, the support and attention elderly individuals with chronic illnesses receive from their families holds significant importance. Korean culture strongly emphasizes family values, interdependence, and filial piety, which further underscores the crucial role of family support for seniors facing chronic health conditions. On the other hand, individualistic tendencies are emphasized in cultures such as the United States, and social support for healthcare can take many forms rather than being family-oriented. Therefore, it is essential to understand the coping mechanisms in conflicts between elderly individuals with chronic diseases and their families in Asian culture.

Previous studies have mainly focused on examining the relationship between high levels of depression and higher family conflict among elderly individuals [15,16,17]. However, there is still a lack of understanding about the moderating effects of self-esteem among the various factors influencing the relationship between depression and family conflict coping strategies of elderly individuals with chronic diseases. Self-esteem refers to positive self-perception, and maintaining and reinforcing it is vital in maintaining a healthy mental state [18]. High stress’s effects on psychological outcomes such as depression tend to be associated with low self-esteem [19]. High self-esteem is considered an internal resource, and positive self-perception can affect interpersonal coping styles [20]. According to stress coping theory, individuals employ various coping strategies when confronted with stressful situations [21,22]. These strategies subsequently shape an individual’s psychological state and behavior [21,22]. Notably, depression is a stressor for chronically ill older adults, highlighting the significance of self-esteem as an intrinsic resource crucial for positive and effective coping in such circumstances [19,20]. High self-esteem levels were positively associated with active coping, planning, and seeking emotional support strategies [23]. Based on these previous studies, high self-esteem is considered an important moderating factor that helps older people not only effectively manage depression even in the presence of chronic diseases but also cope with family conflicts through positive self-awareness. Therefore, self-esteem is believed to be a crucial influencing factor in the relationship between depression and family conflict coping strategies of elderly individuals with chronic diseases.

This study aims to investigate the moderating effects of self-esteem in the relationship between depression and family conflict coping strategies among the elderly with chronic diseases. This research will not only contribute to a theoretical understanding of the association between depression and family conflict coping strategies of elderly individuals with chronic diseases, but also provide a foundation for cultivating strategies to enhance self-esteem in these relationships.

Accordingly, this study aimed to investigate the relationship between depression and family conflict coping strategies in the elderly with chronic diseases and identify the self-esteem moderating effect. Our study encompassed sociodemographic data, depression, self-esteem, and family conflict coping strategies factors. For this study’s participants, the level of depression and family conflict coping strategies among the elderly with chronic diseases may be different from those of the elderly without chronic diseases. The family conflict coping strategy, which is one of the main independent variables, inevitably requires that the target household consists of at least two household members. Therefore, the subjects of this study were the elderly with chronic diseases, excluding single-person households.

Based on the existing literature regarding elderly individuals with chronic illnesses, we anticipated that depression would be related to family conflict coping strategies. Depressed individuals tend to engage in negative thought patterns, leading to self-criticism and reduced self-confidence, ultimately impairing their ability to cope with family conflicts [9,22]. We hypothesized that self-esteem is an important moderating factor in the relationship between specific psychosocial variables such as depression and family conflict coping strategies. According to Lazarus and Folkman’s stress and coping theory, coping strategies involve how individuals respond to specific stressors by thinking, feeling, and behaving to reduce perceived stress and negative emotions [21,22]. As an individual factor, self-esteem is an important prerequisite for coping. High self-esteem is associated with the elderly effectively managing depression [19], even in chronic diseases and coping positively with family conflicts through improved self-awareness [23].

We also compared differences in depression, self-esteem, and coping with family conflict across demographic characteristics. Ultimately, this study aimed to understand family conflict coping strategies in older adults with chronic illnesses and provide insights useful for developing interventions and support programs to improve self-esteem and depression.

## 2. Materials and Methods

### 2.1. Research Design

This is an explanatory study that utilizes secondary data analysis from national data to understand the moderating effect of self-esteem in the relationship between depression and family conflict coping strategies among the elderly with chronic diseases.

### 2.2. Subjects

For this study, data from the 16th Korea Welfare Panel Study (KOWEPS) conducted by the Korea Institute for Health and Social Affairs (KIHASA) and the Social Welfare Institute of Seoul National University (SNU) in 2021 will be used. The Korea Welfare Panel Study has been conducted annually since 2006 and involves direct household visits by surveyors targeting all household members aged 15 and above, excluding middle and high school students. As a suitable panel study for low-income research, approximately 50% of the total sample is allocated to households with less than 60% of the median income, resulting in a higher proportion of low-income households compared to the actual population. The survey covers various topics, including labor activities, economic situations, welfare needs, and social security enrollment status.

**Figure 1:**
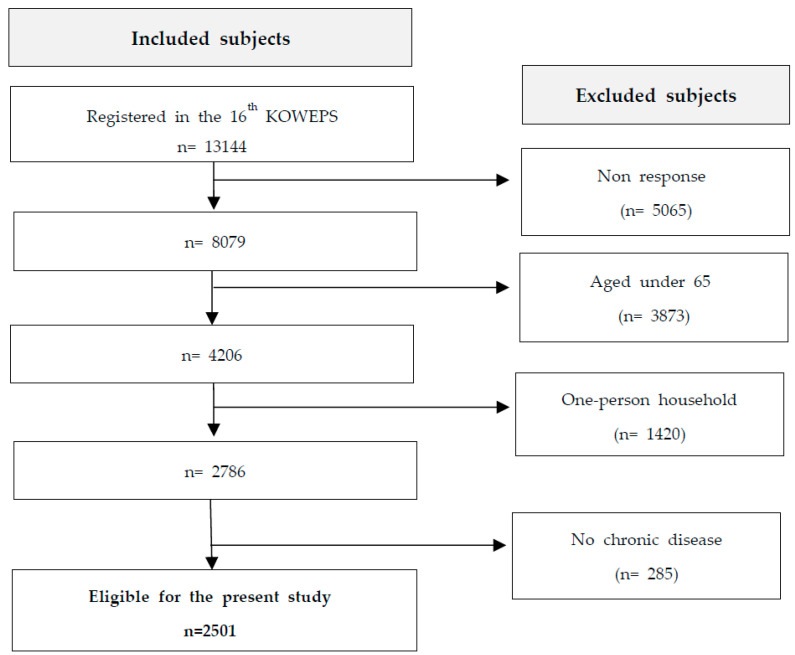
Subject selection process.

The target population for this study consisted of 2501 individuals aged 65 and above and with chronic diseases from 13,144 household members and 5996 households in the 16th KOWEPS. Non-responders, those under 65, one-person households, and participants without chronic diseases were excluded from the analysis (Figure 1).

### 2.3. Measurements

#### 2.3.1. Depression

Depression was measured using the CES-D11. The CES-D11 is an abbreviated version of the CES-D (Center for Epidemiologic Studies Depression Scale), which was initially developed by Radloff (1977) and later adapted for the Korean population by Cho et al. [24]. The reliability and validity of CES-D11 was confirmed in the study by Hoe et al. [25]. This tool consists of 11 items that measure the frequency of depressive symptoms experienced over the past week. Depression is measured on a 4-point scale (1–4 points), with higher total scores indicating higher levels of depression.

The scale includes survey items such as “I did not feel like eating; my appetite was poor.”, “I felt lonely.”, “People were unfriendly.”, and “I could not get going.”. Negative items were reverse scored.

In a study by Kang [26] using the Korea Welfare Panel data, the Cronbach’s alpha for CES-D11 was 0.97; in this study, it was 0.88.

#### 2.3.2. Self-Esteem

Self-esteem was measured using the Rosenberg Self-Esteem Scale. This scale targets the status at the time of the survey and consists of 10 items on a 4-point scale (1–4 points).

The scale includes survey items such as “I feel that I’m a person of worth, at least on an equal plane with others.”, “I take a positive attitude toward myself.”, “I wish I could have more respect for myself.”, and “I certainly feel useless at times.”. Negative items were reverse scored. Higher total scores indicate higher levels of self-esteem.

In a study using the Korea Welfare Panel data by Yang and Kim [27], the Cronbach’s alpha for self-esteem was 0.76; it was 0.75 in this study.

#### 2.3.3. Family Conflict Coping Strategies

The tool of Family Conflict Coping Strategies was developed by the Korea Institute for Health and Social Affairs [28]. It consists of 5 items that measure the coping strategies of individuals regarding family conflicts on a 5-point scale (1–5 points). Higher total scores indicate more positive family conflict coping strategies.

The scale includes survey items such as “My family members always discuss problems calmly.”, and “My family members often reproach each other”. Negative items were reverse scored. Higher total scores indicate more positive family conflict coping strategies.

In a study by Kang [26] using the Korea Welfare Panel data, the Cronbach’s alpha for family conflict coping strategies was 0.82; in this study, it was 0.76.

### 2.4. Data Analysis Method

To compare the differences in depression, family conflict coping strategies, and self-esteem according to the participants’ general characteristics, *t*-tests (degrees of freedom = 2500) or ANOVA were used. The correlation between depression, conflict coping strategies, and self-esteem was examined using Pearson’s correlation coefficient.

To explore the moderating effect of self-esteem in the relationship between depression and conflict coping strategies, a hierarchical regression analysis was conducted. To examine the effect of the interaction between independent variables and control variables on the dependent variable, the study population was divided into four groups, above and below the respective averages of the independent and control variables.

Before conducting regression analysis, mean-centering was performed to check for the multicollinearity in the regression model. The interaction terms used to test the moderating effect were expressed as the product of the independent and control variables, which can lead to unnecessary multicollinearity. In case there exists multicollinearity between the independent and control variables and the interaction terms, mean-centering can be used to eliminate such a factor.

The SPSS Statistics 27.0 program was used to analyze the data of this study.

## 3. Results

### 3.1. Depression, Self-Esteem, and Family Conflict Coping Strategies by General Characteristics

The levels of depression among participants showed significant differences according to gender, age, education level, religious affiliation, and livelihood support status. Depression was higher in females than in males (t = −8.19, *p* < 0.001) and was higher among those aged 80 years and above (t = −7.60, *p* < 0.001). Additionally, individuals with lower education levels had higher levels of depression (F = 48.28, *p* < 0.001), and those without religious affiliation showed higher levels of depression (t = −1.97, *p* = 0.049). Participants receiving livelihood support also exhibited higher levels of depression (t = 9.28, *p* < 0.001).

Self-esteem was higher in males than in females (t = 4.08, *p* < 0.001) and was higher among those aged under 80 years (t = 7.99, *p* < 0.001). Furthermore, self-esteem was higher among individuals with higher education levels (F = 59.51, *p* < 0.001), those with religious affiliation (t = 3.70, *p* < 0.001), and those not receiving livelihood support (t = −10.33, *p* < 0.001).

Family conflict coping strategies showed significant differences in gender, education level, religious affiliation, and livelihood support status. Male participants exhibited more positive coping strategies (t = 3.03, *p* = 0.003), and coping strategies became more positive with higher education levels (F = 8.95, *p* < 0.001). Additionally, participants with religious affiliation (t = 3.46, *p* = 0.001) and those not receiving livelihood support (t = −7.24, *p* < 0.001) showed more positive family conflict coping strategies (Table 1).

### 3.2. Correlations among Depression, Self-Esteem, and Family Conflict Coping Strategies

Depression among participants showed significant negative correlations with self-esteem (r = −0.564, *p* < 0.001) and family conflict coping strategies (r = −0.266, *p* < 0.001). Self-esteem showed a significant positive correlation with family conflict coping strategies (r = 0.307, *p* < 0.001).

### 3.3. Moderating Effect of Self-Esteem in the Relationship between Depression and Family Conflict Coping Strategies

Prior to testing the moderating effect of self-esteem in the relationship between depression and family conflict coping strategies, mean-centering of independent and control variables was conducted. After the process, the variation inflation factor (VIF) between independent variables was found to be less than 10 (1.010–1.765), indicating no multicollinearity issues. The Durbin–Watson statistic for the regression model was 1.289, indicating no autocorrelation in the dependent variable.

Regression models 1 (F = 16.75, *p* < 0.001) and 2 (F = 39.02, *p* <0.001) were statistically significant and showed an increase of 5.3% in the explanatory power of the dependent variable while controlling for gender, education level, religious affiliation, and livelihood support status. In addition, in model 2, depression was found to have a significant effect on family conflict coping strategies (Table 2).

Model 3, which included self-esteem as a control variable, was also statistically significant (F = 47.14, *p* < 0.001) and showed a 3.1% increase in the model*’*s explanatory power. Also, depression (β = −0.13, t = −5.44, *p* < 0.001) and self-esteem (β = 0.22, t = 9.36, *p* < 0.001) significantly influenced family conflict coping strategies.

Model 4 examined the moderating effect of self-esteem in the relationship between depression and family conflict coping strategies. The model was statistically significant (F = 43.20, *p* < 0.001) and showed a 0.5% increase in the explanatory power. This result indicated that self-esteem had a moderating effect, and the effect of depression on family conflict coping strategies was higher when interacting with self-esteem.

**Figure 2:**
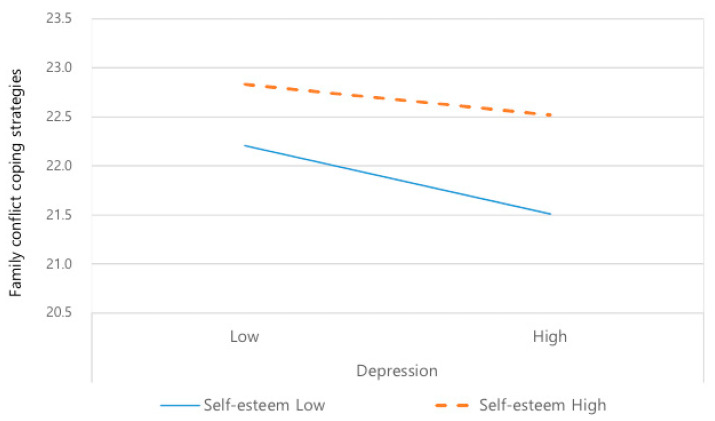
Moderating effect of self-esteem between depression and family conflict coping strategies.

The moderating effect can be observed by dividing the entire sample into four groups based on the means of depression and self-esteem. Figure 2 shows that the influence of depression on family conflict coping strategies differs according to the level of self-esteem (β = 0.09, t = 3.73, *p* < 0.001). Specifically, the change in family conflict coping strategies due to increased depression was more significant in the low self-esteem group than the high self-esteem group (Figure 2).

## 4. Discussion

This study revealed an association between higher levels of depression and negative family conflict coping strategies among elderly individuals with chronic diseases. It is well documented that elderly individuals with chronic diseases experience physical deterioration, social limitations, and financial burdens, which can lead to a sense of loss of control and commonly result in experiencing depression [29,30]. Chronic diseases in the elderly can worsen their health status, impose restrictions on daily activities, and consequently lead to mental health issues such as depression and family conflicts [29,30]. Depression affects negative emotions and thoughts [31]. Moreover, depression can reduce self-efficacy, making it difficult for older adults with chronic illnesses to face and deal with family conflicts and making them lose the confidence needed to overcome and resolve them. Family systems theory assumes that family members’ behaviors are interdependent and influence each other, and one person’s behavior can impact the entire family system [32]. The psychological outcomes of individuals with chronic diseases can also influence interactions within the family [33]. Therefore, depression in elderly individuals with chronic diseases can not only disrupt their daily activities but also affect their interactions with family members [10]. In collectivist and family-centered Asian cultures, the role of family and social support holds significant value. These values can impact older adults’ ability to cope with depression and family conflicts [14]. This result signifies that older adults can find psychological stability and reassurance through the assistance and support of their families, especially when they are experiencing depressive states. Consequently, family support can play a vital role in their ability to navigate family conflicts. To enhance the mental well-being of older adults and improve family relationships, it is crucial to consider strategies for dealing with depression and family conflict within the context of their cultural background.

Living with chronic illness can threaten health and quality of life, with its impact influenced by individual coping strategies and personal resources [22]. According to Lazarus and Folkman’s stress and coping theory, coping strategies involve how individuals respond to specific stressors by thinking, feeling, and behaving to reduce perceived stress and negative emotions [21,22]. Individual factors are aspects related to coping and can be seen as foundational prerequisites for the coping process [21]. As an individual factor, in this study, older males with chronic illness tended to use more positive family conflict coping strategies than older females. Additionally, individuals with higher levels of education were more likely to adopt positive family conflict coping strategies. Further, positive family conflict coping strategies emerged even in the absence of religious affiliation and financial support. In general, people often use both emotion-focused and problem-focused strategies in stressful situations [22]. When faced with depression, elderly individuals with chronic diseases may find it challenging to adopt proactive and problem-solving coping strategies. According to the findings of Féki et al. [34], emotion-focused coping strategies were more prevalent among elderly individuals with high levels of depression. To enhance coping strategies for family conflict among elderly individuals with chronic illnesses, it is crucial to understand individual coping abilities, sociodemographic characteristics, and depression factors. Therefore, healthcare practitioners should assess depression levels, identify family conflict coping strategies, and gauge how effectively elderly individuals with chronic illness perceive and utilize these strategies in certain situations. Additionally, providing support for family members to cope with and resolve conflicts based on the understanding of each other’s emotions are crucial.

On the other hand, it was found that the increase in depression leading to an increase in negative family conflict coping strategies was more significant in the group with lower self-esteem than in the group with higher self-esteem. Low self-esteem is related to limited personal resources to cope with challenging life situations [35], which can lead to maladaptive coping strategies [35,36]. Qualitative research on elderly individuals with chronic diseases has emphasized the importance of learning to live with the disease, accepting chronic diseases as part of life, and valuing one’s existence with self-respect and self-trust [29]. Therefore, enhancing self-esteem in elderly individuals with chronic diseases may improve family conflict coping strategies. High self-esteem can facilitate communication and cooperation among family members and enable better resolution and coping with conflicts within the family. This suggests that participants’ self-esteem level needs to be considered when implementing interventions to strengthen family conflict coping strategies.

Moreover, this study revealed the moderating effect of self-esteem on the relationship between depression and family conflict coping strategies among elderly individuals with chronic diseases. It was found that self-esteem significantly moderates the relationship between depression and family conflict coping strategies. Low self-esteem is associated with vulnerability to psychological stress [37]. Thus, improving self-esteem in elderly individuals with chronic diseases could alleviate depression and regulate family conflicts. High self-esteem in elderly individuals with chronic diseases may facilitate communication and cooperation among family members, leading to better resolution and coping with family conflicts [38,39]. Consequently, healthcare professionals should consider providing family-centered therapy or counseling that can help elderly individuals with chronic diseases understand and accept their situation, value their self-worth, and trust themselves [36,40]. This study found the importance of improving self-esteem through therapeutic and supportive interventions on the relationship between depression and coping with family conflicts in the elderly with chronic diseases. Individual counseling and cognitive behavioral therapy can be practical tools to increase self-esteem [41,42]. Moreover, the role of family members in strengthening the self-esteem of older people with chronic illnesses cannot be overemphasized. This study highlights the pivotal role of self-esteem in the interplay between depression and family conflict coping among elderly individuals with chronic diseases. It underscores the practical implications of interventions that target self-esteem enhancement. Both individual therapeutic approaches and family-focused interventions hold the potential to elevate self-esteem, contributing to improved emotional well-being and adaptive coping strategies for the vulnerable elderly with chronic diseases.

## 5. Limitations

There are several limitations to this study. Firstly, the study focused on elderly individuals with chronic diseases in a specific region, limiting the generalizability of the results. Additional research targeting diverse population groups is necessary to validate these findings. Secondly, the study did not include family environmental variables that may influence family conflict coping strategies, which leaves the possibility of residual confounding factors. Future research should consider exploring the associations between various family environmental factors and coping with family conflicts. Thirdly, this study did not consider the changes in its prime variables, a limitation due to utilizing cross-sectional data. Longitudinal studies will be recommended in the future.

## 6. Conclusions

According to the results of this study, depression in the elderly with chronic diseases was associated with negative family conflict coping strategies. Depression may be related to negative family conflict coping strategies with negative thought patterns, low self-care confidence, and increased dependence on family members. In particular, in Asian cultures that value collectivism and are family-centered, family support and interest in health care for the elderly suffering from chronic diseases and improvement of family conflict coping strategies are essential.

An important result of this study is that self-esteem showed a moderating effect on the relationship between depression and family conflict coping strategies in the elderly with chronic diseases. Self-esteem is associated with an individual’s self-confidence, positive self-perception, and inner stability, which can impact depression and coping with family conflict. When self-esteem is low, older adults might experience diminished positive self-evaluation and weakened stress-coping abilities in the face of difficulties, ultimately exerting negative influences on dealing with depression and managing family conflict. To enhance self-esteem among older adults with chronic illnesses, focusing on recognizing their strengths and abilities, identifying positive traits, managing their health and well-being, learning self-care methods, and building an environment where they can communicate and value themselves through social support from family could be beneficial.

Among the sociodemographic characteristics of older adults with chronic illnesses, gender, educational level, religious affiliation, and the presence of financial support can play a crucial role in an individual’s depression, family conflict coping strategies, and self-esteem. Male older adults with chronic illnesses tended to use positive family conflict coping strategies more than their female counterparts. Individuals with higher levels of education were inclined to adopt positive family conflict coping strategies. Also, positive family conflict coping strategies were shown even in cases where religious affiliation and financial support were absent. These findings emphasize the need for an individualized target education plan that considers individual characteristics to improve coping with family conflicts in the elderly with chronic diseases.

## Figures and Tables

**Table 1:** Depression, self-esteem, and family conflict coping strategies by general characteristics (n = 2501).

General Characteristics	N (%)	Depression	Self-Esteem	Family Conflict Coping Strategies
		M ± SD		
Gender				
Male	1166 (46.6)	14.93 ± 4.74	30.19 ± 4.01	22.49 ± 2.06
Female	1335 (53.4)	16.58 ± 5.28	29.54 ± 3.95	22.22 ± 2.32
t (*p*)		−8.19 (<0.001)	4.08 (<0.001)	3.03 (0.003)
Age				
65–79	1710 (68.4)	15.29 ± 4.91	30.27 ± 3.90	22.40 ± 2.20
Above 80	791 (31.6)	16.93 ± 5.31	28.92 ± 4.03	22.24 ± 2.23
t (*p*)		−7.60 (<0.001)	7.99 (<0.001)	1.61 (0.108)
Education				
Below elementary	1316 (52.6)	16.71 ± 5.34	29.11 ± 4.06	22.18 ± 2.27
Middle and high school	1005 (40.2)	14.94 ± 4.72	30.43 ± 3.84	22.51 ± 2.09
Above college	180 (7.2)	14.01 ± 3.92	31.89 ± 2.98	22.70 ± 2.26
F (*p*)		48.28 (<0.001)	59.51 (<0.001)	8.95 (<0.001)
Religion				
Yes	1361 (54.4)	15.62 ± 4.99	30.11 ± 3.87	22.49 ± 2.15
No	1140 (45.6)	16.03 ± 5.22	29.52 ± 4.12	22.18 ± 2.27
t (*p*)		−1.97 (0.049)	3.70 (<0.001)	3.46 (0.001)
Livelihood Benefits				
Yes	175 (7.0)	19.20 ± 6.07	26.90 ± 4.69	21.19 ± 3.03
No	2326 (93.0)	15.55 ± 4.93	30.06 ± 3.85	22.43 ± 2.11
t (*p*)		9.28 (<0.001)	−10.33 (<0.001)	−7.24 (<0.001)
Range		11–44	11–40	9–25

M = mean; SD = standard deviation.

**Table 2:** Moderating effect of self-esteem.

Independent Variables	Model 1			Model 2			Model 3			Model 4		
	β	t	*p*	β	t	*p*	β	t	*p*	β	t	*p*
Gender ^1^—female	−0.05	−2.14	0.032	−0.02	−0.85	0.397	−0.03	−1.29	0.198	−0.03	−1.54	0.124
Education ^2^												
Middle and high	0.05	2.28	0.022	0.02	0.88	0.379	0.01	0.13	0.901	0.01	0.32	0.749
Above college	0.04	2.03	0.043	0.02	0.92	0.356	−0.01	−0.22	0.824	−0.01	−0.03	0.975
Religion ^3^—yes	0.07	3.72	<0.001	0.06	3.28	0.001	0.05	2.85	0.004	0.06	2.94	0.003
Livelihood benefits ^4^—no	0.14	6.94	<0.001	0.10	5.00	<0.001	0.08	3.93	<0.001	0.07	3.75	<0.001
Depression (A)				−0.24	−12.06	<0.001	−0.13	−5.44	<0.001	−0.08	−3.04	0.002
Self-esteem (B)							0.22	9.36	<0.001	0.20	8.14	<0.001
A × B										0.09	3.73	<0.001
R2	0.033			0.086			0.117			0.122		
F (*p*)	16.75 (<0.001)			39.02 (<0.001)			47.14 (<0.001)			43.20 (<0.001)		

References: ^1^ male, ^2^ elementary school, ^3^ no, ^4^ yes.

## Data Availability

Please contact the corresponding author to obtain the data.

## References

[1] Naja S., Makhlouf M., Chehab M.A.H. (2017). An ageing world of the 21st century: A literature review. Int. J. Community Med. Public Health.

[2] Statistics Korea Major Population Indicators for Future Population Projections. https://kosis.kr/statHtml/statHtml.do?orgId=101&tblId=DT_1BPA002&checkFlag.

[3] Zhao C., Wong L., Zhu Q., Yang H. (2018). Prevalence and correlates of chronic diseases in an elderly population: A community-based survey in haikou. PLoS ONE.

[4] Kang M., Kim J., Bae S.-S., Choi Y.-J., Shin D.-S. (2014). Older adults’ perception of chronic illness management in south korea. J. Prev. Med. Public Health.

[5] Zis P., Daskalaki A., Bountouni I., Sykioti P., Varrassi G., Paladini A. (2017). Depression and chronic pain in the elderly: Links and management challenges. Clin. Interv. Aging.

[6] Zhang Y., Chen Y., Ma L. (2018). Depression and cardiovascular disease in elderly: Current understanding. J. Clin. Neurosci..

[7] Hu T., Zhao X., Wu M., Li Z., Luo L., Yang C., Yang F. (2022). Prevalence of depression in older adults: A systematic review and meta-analysis. Psychiatry Res..

[8] Aziz R., Steffens D.C. (2013). What are the causes of late-life depression?. Psychiatr. Clin. N. Am..

[9] Chang L.Y., Wu S.Y., Chiang C.E., Tsai P.S. (2017). Depression and self-care maintenance in patients with heart failure: A moderated mediation model of self-care confidence and resilience. Eur. J. Cardiovasc. Nurs..

[10] Schulz R., Beach S.R., Czaja S.J., Martire L.M., Monin J.K. (2020). Family caregiving for older adults. Annu. Rev. Psychol..

[11] Lichtenthal W.G., Kissane D.W. (2008). The management of family conflict in palliative care. Prog. Palliat. Care.

[12] El-Slamon M., Al-Moteri M., Plummer V., Alkarani A.S., Ahmed M.G. (2022). Coping strategies and burden dimensions of family caregivers for people diagnosed with obsessive-compulsive disorder. Healthcare.

[13] Mosher C.E., Ott M.A., Hanna N., Jalal S.I., Champion V.L. (2015). Coping with physical and psychological symptoms: A qualitative study of advanced lung cancer patients and their family caregivers. Support. Care Cancer.

[14] Le H., Newman A., Menzies J., Zheng C., Fermelis J. (2020). Work–life balance in asia: A systematic review. Hum. Resour. Manag. Rev..

[15] Park M., Unützer J., Grembowski D. (2014). Ethnic and gender variations in the associations between family cohesion, family conflict, and depression in older asian and latino adults. J. Immigr. Minor. Health.

[16] Wong J.J., Frost N.D., Timko C., Heinz A.J., Cronkite R. (2020). Depression and family arguments: Disentangling reciprocal effects for women and men. Fam. Pract..

[17] Shao M., Chen J., Ma C. (2022). Research on the relationship between chinese elderly health status, social security, and depression. Int. J. Environ. Res. Public Health.

[18] Bergman Y.S. (2022). Ageism and psychological distress in older adults: The moderating role of self-esteem and body image. J. Appl. Gerontol..

[19] Park Y., Park S.-Y., Williams M., Shibusawa T., Martin J.I. (2021). Family conflicts, coping skills, depressive symptoms, and gender among korean american adolescents: Mediating effects of self-esteem. J. Soc. Soc. Work Res..

[20] Yang Y., Sun G., Dong X., Zhang H., Xing C., Liu Y. (2019). Preoperative anxiety in chinese colorectal cancer patients: The role of social support, self-esteem and coping styles. J. Psychosom. Res..

[21] Folkman S. (2010). Stress, coping, and hope. Psycho-Oncology.

[22] Kristofferzon M.L., Engström M., Nilsson A. (2018). Coping mediates the relationship between sense of coherence and mental quality of life in patients with chronic illness: A cross-sectional study. Qual. Life Res..

[23] Skwirczyńska E., Wróblewski O., Tejchman K., Ostrowski P., Serwin N. (2022). Prostate cancer eligible for radical prostatectomy: Self-esteem of patients and forms of coping with stress. Int. J. Environ. Res. Public Health.

[24] Cho M., Kim K. (1993). Diagnostic validity of the ces-d(korean version) in the assessment of dsm-iii-r major depression. J. Korean Neuropsychiatr. Assoc..

[25] Hoe M., Park B., Bae S. (2015). Testing measurement invariance of the 11-item korean version ces-d scale. Ment. Health Soc. Work..

[26] Kang D.H. (2021). Effect of restrictions on the activities of daily living on depression of the elderly: Focused on the moderating effect of family and care worker assistance. J. Korea Contents Assoc..

[27] Yang J., Kim M. (2016). The effect of self-esteem on elderly depression: Moderated mediation effects of family relations satisfaction and working types. J. Fam. Relat..

[28] Lee E., Chae J., Nam S.I. (2018). The relationship among ender role attitudes, depression, and coping with family conflict in older males. J. Korea Gerontol. Soc..

[29] Chiaranai C., Chularee S., Srithongluang S. (2018). Older people living with chronic illness. Geriatr. Nurs..

[30] Zhang C., Chang Y., Yun Q., Lu J., Zheng X., Xue Y., Zhao X., Yuan W., Zou J., Zheng J. (2022). The impact of chronic diseases on depressive symptoms among the older adults: The role of sleep quality and empty nest status. J. Affect. Disord..

[31] Park L.T., Zarate C.A. (2019). Depression in the primary care setting. N. Engl. J. Med..

[32] Haefner J. (2014). An application of bowen family systems theory. Issues Ment. Health Nurs..

[33] Cohen M.S. (1999). Families coping with childhood chronic illness: A research review. Fam. Syst. Health.

[34] Féki I., Turki M., Zitoun I., Sellami R., Baati I., Masmoudi J. (2019). Dépression et stratégies de coping chez les sujets âgés atteints de diabète de type 2. L’encéphale.

[35] Orth U., Robins R.W., Meier L.L. (2009). Disentangling the effects of low self-esteem and stressful events on depression: Findings from three longitudinal studies. J. Pers. Soc. Psychol..

[36] Liu S.Y., Wrosch C., Morin A.J., Quesnel-Vallée A., Pruessner J.C. (2019). Changes in self-esteem and chronic disease across adulthood: A 16-year longitudinal analysis. Soc. Sci. Med..

[37] Wågan F.A., Darvik M.D., Pedersen A.V. (2021). Associations between self-esteem, psychological stress, and the risk of exercise dependence. Int. J. Environ. Res. Public Health.

[38] Barreto Andrade D.M., Montargil Rocha R., Santos Ribeiro I.J. (2020). Depressive symptoms and family functionality in the elderly with diabetes mellitus. Issues Ment. Health Nurs..

[39] Nicolson P., Anderson P. (2003). Quality of life, distress and self-esteem: A focus group study of people with chronic bronchitis. Br. J. Health Psychol..

[40] Siltanen H., Jylhä V., Holopainen A., Paavilainen E. (2019). Family members’ experiences and expectations of self-management counseling while caring for a person with chronic obstructive pulmonary disease: A systematic review of qualitative evidence. JBI Database Syst. Rev. Implement. Rep..

[41] Selçuk-Tosun A., Zincir H. (2019). The effect of a transtheoretical model-based motivational interview on self-efficacy, metabolic control, and health behaviour in adults with type 2 diabetes mellitus: A randomized controlled trial. Int. J. Nurs. Pract..

[42] Mohd-Sidik S., Akhtari-Zavare M., Periasamy U., Rampal L., Fadhilah S.I., Mahmud R. (2018). Effectiveness of chemotherapy counselling on self-esteem and psychological affects among cancer patients in malaysia: Randomized controlled trial. Patient Educ. Couns..

